# Intercalated Nanocomposites Based on High-Temperature Superconducting Ceramics and Their Properties

**DOI:** 10.3390/ma2042154

**Published:** 2009-12-02

**Authors:** Anahit Tonoyan, Christoph Schiсk, Sevan Davtyan

**Affiliations:** 1 Department of Chemistry, State Engineering University of Armenia, 105 Teryan str., 0009 Yerevan, Armenia; E-Mail: atonoyan@seua.am (A.T.); 2 Institute of Physics, University of Rostock, Universitätsplatz 3, 18051 Rostock, Germany; E-Mail: christoph.schick@uni-rostock.de (C.S.)

**Keywords:** polymer-ceramic nanocomposites, superconducting, physical-mechanical, dynamical-mechanical properties, morphological structure

## Abstract

High temperature superconducting (SC) nanocomposites based on SC ceramics and various polymeric binders were prepared. Regardless of the size of the ceramics’ grains, the increase of their amount leads to an increase of resistance to rupture and modulus and a decrease in limiting deformation, whereas an increase in the average ceramic grain size worsens resistance properties. The SC, thermo-chemical, mechanical and dynamic-mechanical properties of the samples were investigated. Superconducting properties of the polymer ceramic nanocomposites are explained by intercalation of macromolecule fragments into the interstitial layer of the ceramics’ grains. This phenomenon leads to a change in the morphological structure of the superconducting nanocomposites.

## 1. Introduction

Beginning with the discovery of high-temperature superconductivity in perovskite systems containing oxygen by Bednorz and Muller [1,2], the literature on this topic has become a veritable avalanche, as works on fabrication of metal [3,4,5,6,7,8,9,10,11,12,13,14] and polymer [15,16,17,18,19,20,21,22,23,24,25,26] binder composites of various geometries possessing SC properties were undertaken, alongside with fundamental research.

In the literature, polymer ceramic compositions using thermoplastic and reactoplastic polymeric matrixes are already described in [15,16,17,18,19,20,21,22,23,24,25,26]. In [15], products were prepared by incineration of a polymeric matrix. Incineration of the organic part, as a rule, is accompanied by oxidation and thermal destruction, which consumes oxygen irreversibly, thus making the ceramic’s grains amorphous. Loss of SC properties of the articles produced via incineration can be explained by this fact. This demands a full circle restoration. It must be noted that the coke formed after incineration has a negative impact on SC properties. Also known are polymer-ceramic compositions designed to protect high-temperature super-conductors against humidity [13,14].

It is known that particulate polymers, as a rule, improve a number of the features of composites (hardness, impact strength, heat resistance, *etc.*). This is mainly related to the formation of a special interfacial layer between the filler and polymeric binder. High-temperature SC polymer–ceramic composites can be obtained both by the conventional hot pressing of a mixture of ceramic and some ready-made high-molecular weight binder, and by the polymerization filler method [21]. Hot pressing [18,19,20,22,23,24,25,26,27,28] of the Y_1_Ba_2_Cu_3_O_7−*x*_ oxide ceramic and superhigh-molecular polyethylene mixture at 200 °C destroys the SC properties, which are restored only after treatment of samples in a dry oxygen stream [22].

With regard to conventional fillers, several specific properties of the perovskite high-temperature superconductors (layered structure, developed surface of the ceramic grains, catalytic properties, free oxygen dislocated on the surface of the ceramic grains, *etc.*) will have an significant impact, not only on the formation of a phase boundary, and, consequently, on the physical–mechanical properties, but on the SC properties of the polymer–ceramic composites as well. Regardless of the nature of the binder, the critical transition temperature (*T*_c_) into the SC state of polymer–ceramic composites increases by 1–3 K. This increase of the transition temperature is due to the interaction of the polymer chains with the surface of the ceramic grains. One could expect that such an interaction should change the packing and structure of the polymer chains, as well as the conformation at the interphase. In this part of the work interphase phenomena at the ceramic-polymer boundary are investigated for superhigh molecular weight polyethylene + Y_1_Ba_2_Cu_3_O_7-x_ ceramic. The influence of crystalline binders on the valence state of Cu^2+^ (1) in the ceramic has been investigated too.

It is interesting to study the influence of the environment on the superconducting properties of SC ceramics and polymer-ceramic compositions at ambient temperatures. It is known that the bulk oxygen content determines the properties of the high-temperature oxide ceramics of the Y-Ba-Cu-O system, and the pattern in which the oxygen fills the crystallographic positions. Presence of an unstable phase is a feature of present generation superconducting ceramics. Non-stability of the lattice structure is linked with the deficiency of the atoms of oxygen in the elementary cell, accompanied by the accumulation of regular patterns of vacancies in the Cu-O atom chains. Therefore, one can’t deny that the increase of non-stability of the lattice structure of the Y_1_Ba_2_Cu_3_O_7-x_ ceramic over time can force to change its super conducting characteristics, e.g., the critical temperature of transition into the super conducting state (*T_i_*, K); the width of transition (Δ*T_c_*, K); the orthorhombic distortion of the lattice structure ((*b* − *a*)/*a*,*η*).

Taking all this into account, investigations were carried out over a period of one year to determine the change of the superconducting characteristics of the ceramics and of the obtained high-temperature superconducting polymer-ceramic compositions by various methods.

## 2. Experimentation and Characteristics of the Initial Compounds

Powders of the corresponding polymer and Y_1_Ba_2_Cu_3_O_7-*x*_ ceramic were preliminarily blended in an agate mill to give a homogeneous mixture for the formation of items (plate, rod, tube, ring, *etc.*). These pre-made mixtures were then filled into previously heated (130, 150, 160, 200 °C) forms and pressed at 100 mPa for 5, 10, 20, or 30 min.

For the formation of polymer-ceramic SC items from Y_1_Ba_2_Cu_3_O_7-*x*_ ceramic powder at ambient temperature, specimens were pressed with follow-up imbibing with methyl methacrylate, both with initiator (azobisisobutyronitrile) and without, in another series of experiments. To counterbalance vaporization of the monomer, the specimens were placed in sealed glass forms and polymerized at 60–80 °C. As a control of the reaction end, other specimens were tested concomitantly in a DAK-11 microcalorimeter.

For the gas-phase polymerization of ethylene influenced by Y_1_Ba_2_Cu_3_O_6.97_ (particle size < 50 μm), its surface was activated at 197 °C during four hours, then cooled down to ambient temperatures in a dry air environment. Part of the thus obtained ceramic was used for the determination of the critical transition temperature to the superconducting state, whereas the other part was placed in the polymerization reactor. At room temperatures, under a pressure of 20 bar, ethylene was introduced into the reactor filled with hexane, and alkyl aluminum was injected under vigorous agitation (rotation speed: 100 rpm). Ethylene was consumed for three hours, which is a major sign of ethylene polymerization.

Structures of the high-temperature superconducting (HTSC) ceramic and its compositions were determined by X-ray analysis on a DRON 2.0 instrument (λ_CuKr_) in the 15° ≤ 20 ≤ 130° range of angles at room temperature. The critical temperatures of the SC-transitions were measured by the αC-magnetic susceptibility method at the 1 kHz frequency when the amplitude of magnetic field was 10 mE. The physico-mechanical properties were determined on an INSTRON rupture machine. Thermo-oxidation destruction was monitored by a derivatographic method on a MOM brand Q-1500 instrument.

Superconductive composite samples based on super-high-molecular polyethylene (SHMPE) have the form of 3 × 1 × 0.1 cm^3^ plates (matrix:filler = 100:0; 85:15; 50:50; 15:85 mass ratios). These samples were used for studying the dynamic mechanical properties.

The mechanical relaxation properties of the SHMPE composites have been measured using a Du Pont DMA instrument under 0.1, 0.2 mm oscillation amplitude. Structural peculiarities of superconductive polymer-ceramic composites have been investigated using a "Tesla" electron-microscope.

The kinetics of polymerization was investigated on micro-calorimeter DAK-11 in the presence of Y_1_Ba_2_Cu_3_O_6.97_ ceramic. Mn, Zn, Co, Ni containing metal-monomers were synthesized according to literature methods [27].

The frontal polymerization [28] of metal-monomers was carried out in vertically placed glass ampoules. For this purpose, ceramic and metal-monomer powders were mixed into a homogenous mixture in an agate mill and filled in glass ampoule with an inner diameter of 0.8 cm. The samples of SC-ceramics and polymer-ceramic compositions were kept in air at ambient temperatures. Enthalpy of the filled crystalline polyethylene was determined on a DSM-3A differential scanning calorimeter.

Quinol ether [*O,O*-(bis-(1,3,5-tri-*tert*-butyl-4-oxocyclohexadiene-2,5-yl)-2-methyl-5-propylbenzo-quinone dioxime, 1% by mass of divinyl rubber) was added to stitched divinyl rubber (DR) to obtain the corresponding composite specimens. The blend was well mixed with the ceramic and the obtained mixture was polymerized at 200 °C under the impact of a specific pressure (100 mPa) during 30 min. Thus produced plates (1.5 mm thick) were cut into blades, which were tested on an INSTRON rupture machine. Five measurements were taken for each specimen composition, which later were averaged as physical-mechanical characteristics.

In other series of experiments, fractionation of Y_1_Ba_2_Cu_3_O_7-x_ ceramic on diffuser–confuser sieves elaborated at the Institute of Chemical Physics of the Russian Science Academy was examined. The density of various fractions of the powders was determined on hydrostatic scales, while the BET method was used for the determination of the specific surface area. DR initial rubber had a molecular mass of 10^6^ and a polydispersity distribution of 2.5. Average particle size fractionation in between 5 to 30 μm was used in this work.

The used Y_1_Ba_2_Cu_3_O_7-x_ ceramic was of two compositions—Y_1_Ba_2_Cu_3_O_6.97_ and Y_1_Ba_2_Cu_3_O_6.92_—with the following characteristics: superconducting critical transition initiation temperature (*T_c_*)—93 K and 91.5 K, while the width (Δ*T*) was 6.5 and 6.0.

For the formation of polymer-ceramic compositions by the hot-pressing method the following polymeric binders were used: SHMPE *T_melt_* = 128–135 °C and ramified polyethylene (RPE) with *T_melt_* = 105–108 °C, isotactic polypropylene (iPP) with *T_melt_* = 167–171 °C, polybutene (PB) *T_melt_* = 135 °C, co-polymer of ethylene with tetrafluoroethylene (brand name—F-40) *T_melt_* = 265–278 °C, polyvinilidenefluoride (PVF) *T_melt_* = 171–180 °C, polyvinyl alcohol (PVA) with *T_g_* = 85 °C, polyformaldehyde (PFA), polymethylmetacrylate (PMA) with *T_g_*= 100–105 °C, polystyrene (PS) with *T_g_* = 98–102 °C, as well as copolymers of styrene (ST) with methyl metacrylate (MMA) (ST content 80, 60 and 40 mol/%), divinyl rubber (DR) with average molecular mass equal to 106 and 2.5 polydispersity. All the polymers used in this work were fine powders. As an antioxidant, Irganox® 1010 and NG-2246 in 5% weight ratio *vs.* the polymeric binder was used.

## 3. Superconducting Properties of the Polymer-Ceramic Nanocomposites Obtained by the Hot-Pressing Method

**Figure 1:**
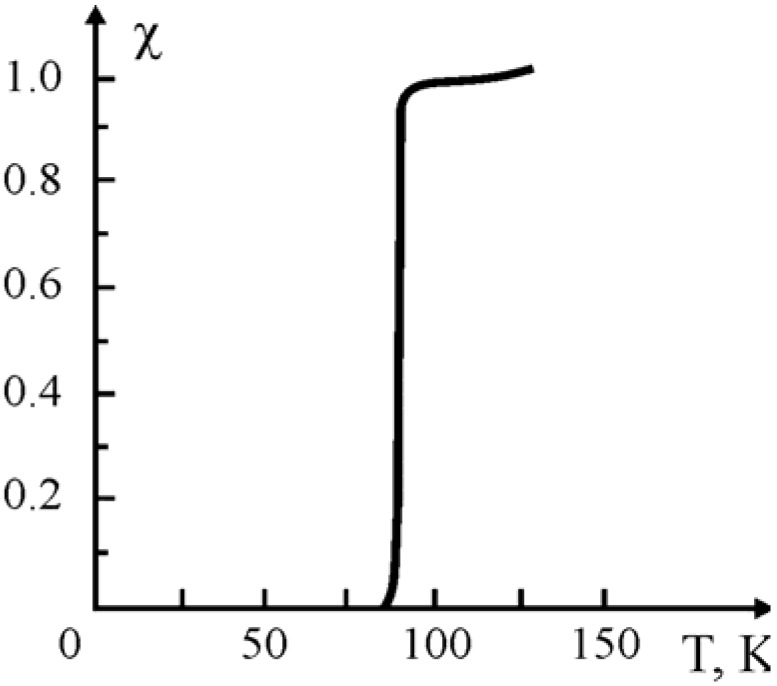
SC transition for the Y_1_Ba_2_Cu_3_O_7-x_ composite with SHMPE. Ceramic/binder weight ratio: 80:20, pressing temperature: 200 °C [18].

It had to be stated that the polymer-ceramic compositions with various binders used exhibit no superconducting properties right after hot pressing at 200 °C during 30 min., e.g., the Meisner effect is missing. Absence of SC properties can be explained by the depletion of oxygen from the orthorhombic super-conducting phase of the ceramic Y_1_Ba_2_Cu_3_O_7-x_ after pressing at 200 °C. The released oxygen interacts irreversibly with the polymeric binder causing subsequent thermo-oxidative destruction of the latter. Actually, the investigations carried out on the compositions with super high molecular polyethylene with the “MOM 1500” derivatographic instrument show that at 160 °C and higher temperatures weight loss took place, which indicates that macromolecules of the binder decompose under thermo-oxidative conditions. Nevertheless, one would hardly expect that only part of the oxygen participates in the thermo-oxidative destruction of the binder, desorbing and diffusing into the polymeric phase from the nucleus of the ceramic’s grains. It seems thus that free oxygen dislocated on the surface of the grains of the oxide ceramic reacts with the polymeric phase. Indirect poof of this assumption comes from restoration of the superconducting properties of the compositions (see Table 1) under an atmosphere of dry oxygen at the α transition temperatures of the polymeric binders. The characteristic curve of the SC transition [Y_1_Ba_2_Cu_3_O_7-x_ + super high molecular polyethylene)] obtained for the samples after the restoration, is given in Figure 1.

Sound proof of the assumption made above is the results of the experiments carried out when Irganox® 1010—a polymeric antioxidant—was added to the initial mixture of composition. As it is known [29,30,31], antioxidant additives in the polymeric matrix substantially reduce the rate of oxidative destruction of the polymers.

Consequently, one can deduce that in this particular case, introduction of minute quantities (0.5 weight% of binder) of antioxidant in the polymer-ceramic compositions decreases the rate of the oxidation reactions and, consequently retards the depletion of the oxygen dislocated on the surface of the ceramic’s grain. Actually, in the compositions having small amounts of antioxidant, right after pressing and without the restoration stage, the samples possess super-conducting properties as follows from the data given in Table 1. In compositions containing antioxidant and without it, when the nature and quantity of the polymer stays intact, the super-conducting transition critical temperature (*T_c_*) stays practically unchanged, whereas the transition end (*T_f_*) changes. In the samples without antioxidants, a wider (*T_c_–T_f_*) transition temperature span seems linked to a non-homogeneous distribution of the oxygen dislocated on the surface of the ceramic’s grain after the stage of restoration.

**Table 1:** Super-conducting properties of the polymer-ceramic nanocomposites after restoration.

Composition	Ceramic/binder weight ratio	T_c_ (K)	T_f_ (K)	Notes
**SHMPE+Y_1_Ba_2_Cu_3_O_7-x_**	80:20	96	84	Obtained after the restoration stage
	85:15	96	84	
	85:15	96	84	
**RPE+ Y_1_Ba_2_Cu_3_O_7-x_**	80:20	94	80	-
**F-40+Y_1_Ba_2_Cu_3_O_7-x_**	75:25	96	77	-
**PB+Y_1_Ba_2_Cu_3_O_7-x_**	80:20	96	83	-
**PVF+Y_1_Ba_2_Cu_3_O_7-x_**	85:15	90	75	-
**PFA+Y_1_Ba_2_Cu_3_O_7-x_**	80:20	88	76	-
**SHMPE +Irganox+Y_1_Ba_2_Cu_3_O_7-x_**	80:20	96	89	Obtained without restoration
**RPE+Irganox +Y_1_Ba_2_Cu_3_O_7-x_**	80:20	94	85	-
**PVA+ Irganox +Y_1_Ba_2_Cu_3_O_7-x_**	85:15	90	80	-

Juxtaposition of the superconducting properties (see Table 1) of the polymer-ceramic compositions with various binders shows that both the beginning and the end of the transition depend considerably upon the nature and chemical composition of the polymeric binder. For the superconducting polymer-ceramic composites, the superconducting critical temperature increases by 2–3 K, except for PVA, PMMA and PFA, as follows from the Table data. It is supposed that this *T_c_* temperature elevation can be ascribed to the physical interaction of separate fragments of the macromolecules of the binder with the surface of the Y_1_Ba_2_Cu_3_O_7-x_ ceramic, up to intercalation of these fragments into the interstitial layer of the ceramic’s grain or their anchoring in free oxygen positions. For the compositions with polyvinyl alcohol the observed decrease of *T_c_* by five degrees speaks to the possible interaction of some fragments of the polymeric chain with the surface of the ceramic. In this case OH groups, like in water, alcohol or weak acids [32], while reacting distort the orthorhombic phase in nearby-surface layers of the ceramic’s grain, and thus decreasing the critical temperature of the superconducting transition. For the polyformaldehyde composition, the considerable *T_c_* decrease can be explained by the high inclination of polyformaldehyde towards thermo-oxidative destruction, which enhances the consumption of oxygen in the ceramic. Moreover, even at the comparatively lower temperature [30] of thermo-oxidation of polyformaldehyde, it was accompanied by a de-polymerization reaction with the release of gaseous formaldehyde, which—in turn—can adversely affect the superconducting properties of the nanocomposites.

## 4. Influence of the Thermal Regimes on the Superconducting Properties of the Polymer-Ceramic Nanocomposites

To ensure steady contact between the polymeric binder and the grains of the ceramic, hot pressing of the mixtures is to be performed at temperatures slightly exceeding the melting or vitrification temperature of the polymeric matrix. The temperature of vitrification of the used acryl and vinyl homo- and copolymers didn’t exceed 100 °C, while the melting temperature of the polyolefins is 120–125 °C (apart from PP, for which *T_melt_* = 170–175 °C). This is why most samples were pressed at 130 °C.

After pressing at 130 °C, samples sustain their superconducting properties, as opposed to the previous case. Nevertheless, the samples based on polyethylene possess the same values of *T_c_* and Δ*T_c_* as the initial ceramic has, whereas for the acryl and vinyl homo- and co-polymers, the initial temperature of transition to the super-conducting properties is 1–2 K higher (Table 2).

**Table 2:** SC characteristics of the nanocomposites based on Y_1_Ba_2_Cu_3_O_6,97_ ceramic (*t_press._*=130 °C, *τ_press._*= 4 min).

Binder in the composition	Content of composition in the binder (weight%)	Antioxidant (5% by weight of binder)	T_c_ (K)	ΔΤ_c_ (Κ)	Difference of the parameters of the elementary cell (A°)	Index of oxygen
**HDPE**	10		92.1	7	0.0732	6.98
	15		91.8	7	0.0719	6.97
**SHMPE**	10	Irganox 1010 NG-2246	91.2	7	0.066	6.93
	15		91.5	~5.0	0.0703	6.96
	20		91.8	6	0.0719	6.97
	15		91.7	6	0.0719	6.97
	15		91.2	6	0.0708	6.96
**PMMA**	10	NG-2246	92.3	8	0.0707	6.96
	15		93.7	7	0.071	6.96
	20		91.7	7	0.0689	6.95
	15		93.2	6	0.0747	6.98
**PS**	10	NG-2246	92	6	0.0719	6.97
	15		93.1	7	0.0752	6.99
	20		92.3	8	0.0703	6.96
	15		93	7	0.0751	6.99
**SPL Styrene-MMA (60:40 Mole%)**	10	NG-2246	91.7	7	0.0689	6.95
	15		92.3	7	0.0659	6.93
	20		92.1	7	0.0666	6.93
	15		92	7	0.0659	6.93
**SPL Styrene-MMA (80:20 Mole%)**	10	NG-2246	92.1	8	0.0689	6.95
	15		91.9	7	0.0707	6.96
	20		92.6	8	0.0739	6.98
	15		93.4	~9.0	0.0767	6.998

There is no distinct relationship between the amount of the ceramic in the composition, the values of *T_c_* and *ΔT_c_* for the interval of concern, and the degree of filling of the superconducting polymer-ceramic materials (80–90 weight%).

Increase of the press load of the compositions up to 200 MPa, doesn’t change the *T_c_* and Δ*T_c_* values. Addition of the antioxidants (5% of the mass of binder) at the given pressing temperature of the composition, practically doesn’t affect either the ceramic’s crystalline lattice structure, or the critical temperatures of transition to the superconducting state.

As it was indicated above, while pressing the polymer-ceramic compositions at 200 °C for 30 min, the superconducting properties of the materials are lost. Cutting the pressing duration down to 4 min at the same temperature, for the compositions based on SHMPE (the content of ceramic is 85% weight) retains the same critical superconducting transition parameters as the initial ceramic has (*T_c_* = 92 K, and Δ*T_c_* = 8 K). Another picture was obtained for PP under the same conditions and at 180 °C: *T_c_* remains constant (91.6 K), while the transition width increases sharply (Δ*T_c_* ≥ 8 K). It is known that the presence of tertiary atoms of carbon in the macro-chain weakens carbon-carbon bonds in that polymer, which makes it less thermo-stable than polyethylene [31]. It is assumed that this inclination of the PP to decompose under thermo-oxidation conditions and participation of oxygen from the super-conducting orthorhombic phase in this process are the main cause of the observed facts.

For the conformation of this supposition, antioxidants (Irganox® 1010, NG-2246) were additionally introduced in the matrix, which should decrease the thermo-oxidative destruction rate of the polymer substantially. Actually, addition of Irganox®1010 contracts the width of the temperature transition into the superconducting state up to the characteristic values of initial ceramic (Δ*T_c_* ≈ 8 K), whereas NG-2246 does not affect the Δ*T_c_* of the compositions based on PP. This is because NG-2246 is not an effective antioxidant for the polyolefins.

Elevation of the pressing temperature of the compositions based on PMMA (the content of the ceramic is 85% in weight) up to 160 °C and higher (duration—15 min, cooling down to 40 °C) increases the Δ*T_c_* appreciably (≥8 K), while the *T_c_* remains steady (91.9–92.3 K).

When NG-2246 antioxidant is added or when the formation duration is cut down to the conventional time of 4 min, then the superconducting characteristics (*T_c_* = 91.5 K and Δ*T_c_* = 8 K) were restored.

Thus, all these results (Table 2) can be explained by the competing action of two processes taking place in parallel:

- interaction of separate elements or fragments of the macromolecule of polymeric binder with the surface of the ceramic’s grain before their intercalation into the interstitial layer of the ceramic;
- thermo-oxidative destruction of the polymeric binder.

In this case, the factors which promote the interaction of the elements of the macromolecule of the binder with the surface of the ceramic (flexibility of the macro chains, elevation of the temperature, *etc.*), as well as the decrease of the rate of thermo-oxidative destruction (reducing the temperature of pressing, introduction of antioxidants), promote or at least maintain the critical superconducting properties which they possess in initial state. Supposedly, oxygen contained in the ceramic’s grains plays an active role in the process of thermo-oxidative destruction of the polymeric matrix.

Nevertheless, to determine whether the thermo-oxidative destruction of the polymeric binder is a governing factor responsible for the widening of Δ*T_c_*, the thermo-chemical properties of the non-filled polymeric binder, as well as of the compositions based on them was investigated.

## 5. Influence of Thermo-Oxidative Destruction on the Superconducting Properties of the Polymer-Ceramic Nanocomposites

**Figure 2:**
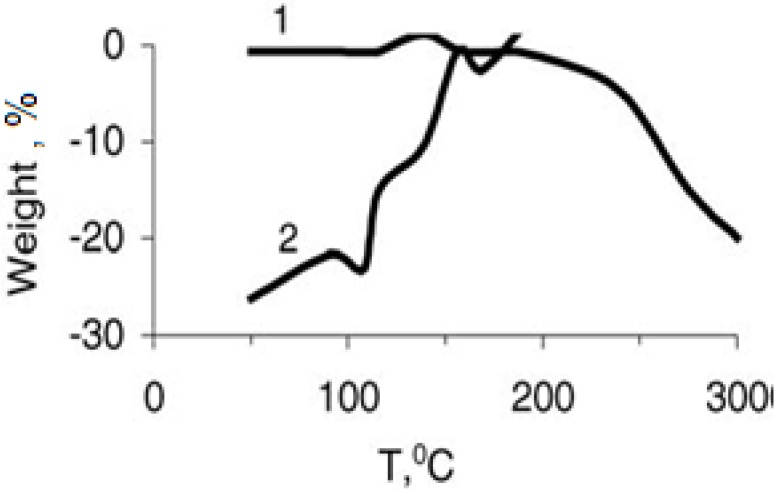
Weight loss (% of the SHMPE) (curve 1) and differential thermal assay (curve 2) via thermo-oxidative destruction of the SHMPE. Heating rate is 3.2 °C/min [19].

**Figure 3:**
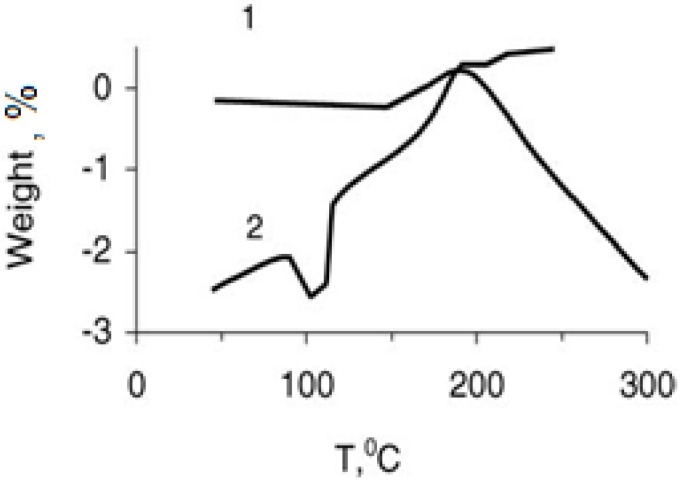
Weight loss (% of the binder) (curve 1) and differential thermal assay (curve 2) of the SHMPE +Y_1_Ba_2_Cu_3_O_6.97_. The composition of the composite: SHMP:Y_1_Ba_2_Cu_3_O_6.97_ = 20:80 (in mass ratio of the monomer). Heating rate is 3.2 °C/min [19].

Results of the derivatographic analysis of the samples under investigation are presented in Table 3 and Figure 2 and Figure 3. The obtained results prove that the Y_1_Ba_2_Cu_3_O_6,.97_ oxide ceramic is stable up to 300 °C, as follows from the thermo-gravimetric curves (TG), and differential thermal analysis (DTA). No displacement of apexes was observed.

Study of the derivatogramms of the non-filled SHMPE (Figure 2), and the compositions based on it (Figure 3), allows us to conclude that in between 150 and 195 °C the exothermic peaks on the DTA curves supposedly are conditioned by the oxidation of the matrix at these temperatures. Furthermore, this conclusion is substantiated by the weight increase of the samples observed on the TG curves. The temperature of initiation of the oxidation of SHMPE in the compositions (Figure 3) is lowered by 10–15 °C with regard to non-filled polymer. Beginning from 185 to 195 °C, thermo-oxidative destruction of the compositions initiates, which is accompanied by the loss of weight of the samples. This means that the formation of the compositions with a SHMPE binder at 200 °C is accompanied by thermo-oxidative destruction of the matrix. PMMA—superconducting ceramic compositions are thermally less stable. Introduction of the superconducting ceramic (filling rate—85% by weight) decreases the temperature of initialization of thermo-oxidation destruction of PMMA from 170 °C down to 155 °C.

**Table 3:** Thermo stability of the polymeric binders and of SC nanocomposites based on them (*t_press._*=130 °C, *τ_press._*= 4 min).

Type of polymeric binder in the composition	Binder content in the composition (in weight%)	Type of used antioxidant (5% of the mass of the binder)	Thermo oxidation destruction initiation temperature (°C)	Weight loss at 300 °C (calc. On the polymeric mass) (mass%)	Notes
**SHMPE**	100		195	2.3	The used ceramic has a low oxygen index and a wide transition into the SC state temperature interval
	10		185	~33.0	
	15		190	~21.0	
	20		195	~20.0	
**PMMA**	100	NG-2246	170	21	Conventional ceramic is used - Y_1_Ba_2_Cu_3_O_6,97_
	15		155	33	
	15		235	21	
**SPL Styrene-MMA (60:40 Mole%)**	100	NG-2246	165	20	-
	15		125	-	-
	15		220	~5	-

Addition of NG-2246 as an antioxidant decreases the rate of thermo-oxidative processes occurring in the matrix, thus increasing the temperature of initiation of destruction of the binder up to 235 °C. It was thought that the presented results fully support the presumption made above and correlate well with the observed peculiarities of the superconducting characteristics of the PMMA-ceramic compositions. The compositions based on styrene with methyl methacrylate (Table 3) are similarly effective in the presence of NG-2246 antioxidant.

## 6. Polymerization of Methyl Methacrylate in the Presence of Y_1_BA_2_CU_3_O_7-X_ Ceramic

**Figure 4:**
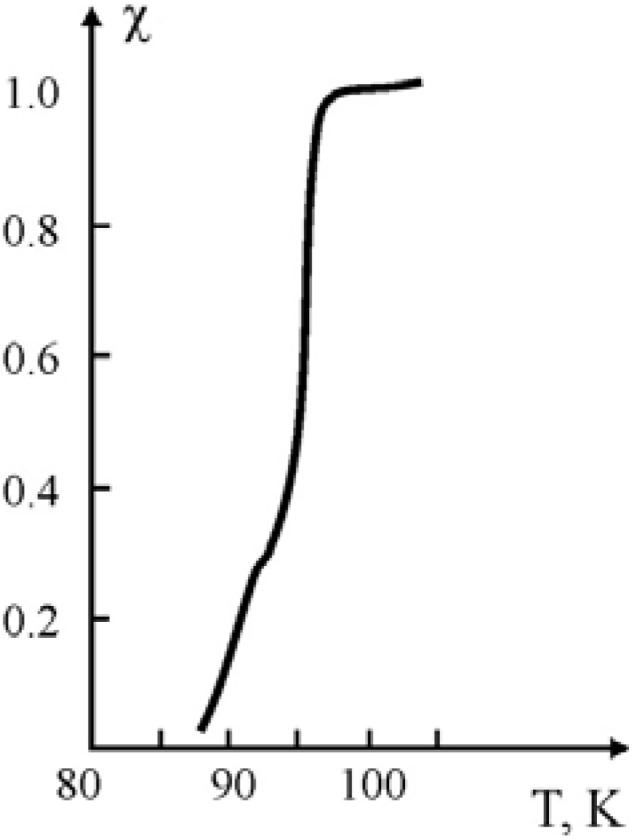
The curve of SC transition for the composition obtained during polymerization of MMA in the pressed samples of the Y_1_Ba_2_Cu_3_O_7-x_ ceramic at 80 °C. Ceramic/binder weight ratio: 88:12 [21].

For the compositions obtained by the polymerization of methyl methacrylate (MMA), in the pressed samples of Y_1_Ba_2_Cu_3_O_7-x_ ceramic, measurement of their superconducting characteristics show that for all the samples the Meisner effect was observed, without the restoration stage. The character of the transition to the super-conducting state for the compositions with methyl methacrylate binder obtained through polymerization filling is given in Figure 4.

**Figure 5:**
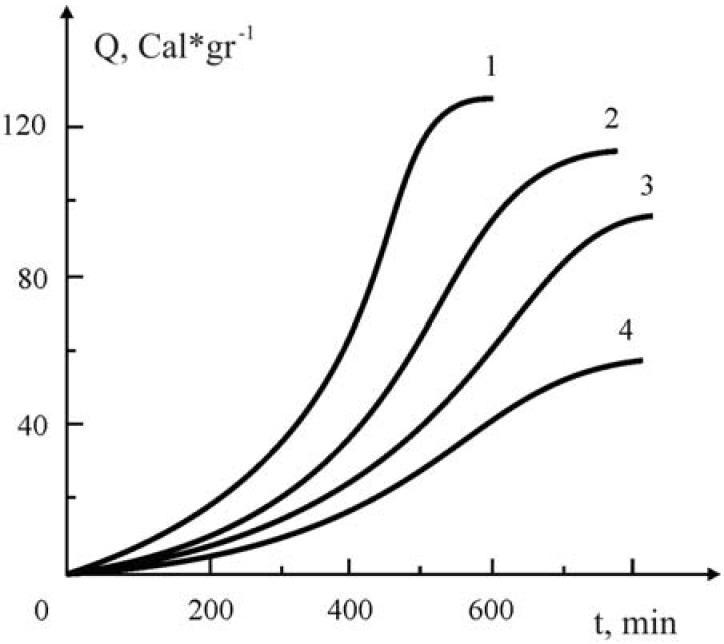
The influence of the added Y_1_Ba_2_Cu_3_O_7-x_ ceramic on the kinetics of polymerization of MMA under the impact of 3·10^-2^ M·L^-1^ at 60 ° C. Y_1_Ba_2_Cu_3_O_7-x_ Ceramic content (in gr.): 0-(1); 0.1(2); 0.2 (3); 0.3 (4) [19].

**Figure 6:**
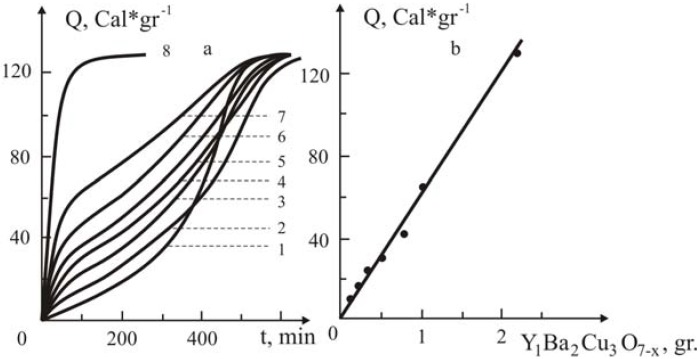
The influence of the added Y_1_Ba_2_Cu_3_O_7-x_ ceramic on the kinetics of polymerization of MMA without initiator at 60°C (a) and the dependence of Q, *vs.* the added ceramic (b). Y_1_Ba_2_Cu_3_O_7-x_ Ceramic content (in gr.): 0(1), 0.1 (2); 0.2 (3); 0.3 (4); 0.5 (5); 0.7 (6); 1 (7); 3.8 (8). Concentration of azobisisobutyronitrile is 3·10^-2^ M´L^-1^ without ceramic [19].

Unexpected results were obtained while studying the influence of the Y_1_Ba_2_Cu_3_O_7-x_ oxide ceramic on the kinetics of the polymerization of methyl methacrylate in the presence and absence of azobisisobutyronitrile (AIBN). As it turned out, for long periods (around 4–5 hours) no polymerization takes place, whereas in the absence of initiator polymerization ocurrs quite quickly when the filling rate was higher [Y_1_Ba_2_Cu_3_O_7-x_ MMA + AIBN = 90:10; 85:15,% by weight]. For the elucidation of such kind of anomalies, the influence of various quantities of the Y_1_Ba_2_Cu_3_O_7-x_ ceramic on the kinetics of the polymerization of MMA in the presence and absence of AIBN was specifically investigated. A series of kinetic curves showing the influence of Y_1_Ba_2_Cu_3_O_7-x_ ceramic on the polymerization of MMA in the presence and absence of ceramic is presented in Figure 5 and Figure 6a, respectively.

From kinetic curve 1 (Figure 5) it follows that in the absence of ceramic, the kinetics of the initiated polymerization of MMA has a conventional trend. The maximal heat evolution is about 130 kcal/degree, which corresponds to the heat effect of MMA polymerization. Addition of the oxide ceramic Y_1_Ba_2_Cu_3_O_7-x_ (Figure 6, curves 2–5) decreases not only the rate of polymerization, but the marginal rate of conversion as well.

A different picture is observed when the polymerization of MMA is carried out in the absence of AIBN. As follows from Figure 6a, in this case the rate of polymerization somehow increases non-conventionally. For this particular case addition of the ceramic, a considerable increase in the rate of polymerization at the initial stages of the process and a corresponding decrease in the gel-effect in the later stages of conversion are noted. These indicated anomalies become conspicuous when the amount of the added ceramic is increased in the initial reaction mixture.

The observed character of the change of the kinetics of polymerization of methyl methacrylate is understandable from the point of view that supposedly some specific interaction did take place for the ceramic both with the monomer, as well as with the initiator. It is known [33] that perovskites of the Y_1_Ba_2_Cu_3_O_7-x_ type possess catalytic properties for numerous chemical reactions. For this case, one could conceive that some separate localities of the ceramic’s grain can interact with MMA and AIBN. In this instance, if the rate of the interaction of the ceramic with the initiator is far higher than with monomer, then some part of the initiator interacts with the active centers of the Y_1_Ba_2_Cu_3_O_7-x_ grains’ surface, and fills them fully, thus excluding the possibility of interaction with MMA. Thus, some part of the initiator is “blocked” by the ceramic’s surface and takes no part in the initiation reaction. For this case, increase of the amount of the added ceramic enhances the share of the “blocked” initiator, and, consequently, reduces the rate of polymerization (Figure 6a, curves 2-5). As to the kinetics of polymerization obtained in the absence of AIBN, here, it seems, the monomer interacts with the active localities of the ceramic’s grain, leading to the formation of primary active centers of polymerization, which are fixed on the solid Y_1_Ba_2_Cu_3_O_7-x_ surface. It is hard to apply the conventional mechanisms of growth and rupture of the chains for this case, because non-mobility of the ends of the macromolecules—capable of participatng in the reactions—excludes the possibility of chain quenching. From this it the sharp increase of the rate of polymerization at the initial fast stages of the reaction becomes understandable. As to the mechanism of the growth of the chains, the monomer reacts with an active center while approaching it. It is apparent that elongation of the macromolecule captures these centers, and, consequently diminishes the accessibility of the centers to monomers. Finally, the kinetics of the chains’ growth reaction on the ceramic’s surface turns into diffusion control and hence the rate of polymerization decreases.

The second region of the kinetic curves (Figure 6a, curves 2-7), where a sharp decrease of the polymerization rate and stepwise increase is observed, is linked with polymerization that occurs, not on the surface, but in the bulk monomer, e.g., the layer of macromolecules that upon reaching a definite dimension encompasses the active chain and partially desorbs from the surface of the ceramic, thus uncovering the active end of the chain. When these uncovered ends accumulate further, the rate of the homogeneous polymerization increases up to complete utilization of the monomer. It’s worth to state that the rate of bimolecular chain termination in the bulk is low because the furled and uncovered macro chains have little mobility.

For this mechanism of chain initiation and growth, it is obvious that an increase of the amount of the added ceramic will bring about the enhancement of the conversion in the first region, where high rates of polymerization occur and a decrease of the latter in the second region is observed (Figure 6a, curves 2-8). It is interesting to note that the extent of conversion, which is reached in the first region of the kinetic curves, is linearly dependent upon the amount of added ceramic (Figure 6b). From the data of this Figure, the quantity of the ceramic which is needed to achieve complete conversion into polymer in the region where the high rate of polymerization occurs was estimated. As expected (Figure 6a, curve 8), addition of 90% by weight of Y_1_Ba_2_Cu_3_O_7-x_ ceramic assures completeness of polymerization in the active polymerization centers on the surface of the ceramic.

The mechanism and topochemistry of the process can be expressed otherwise if the active polymerization centers formed and fixed on the surface of the superconducting ceramic’s grains are of radical type. Initiation of the polymerization (in the absence of AIBN) begins from the surface of the ceramic when the content of filler in the reaction mixture is too high (90% by weight); the polymerization process is characterized by a high rate, and, presumably, is localized at the ceramic-monomer interstitial boundary.

It is known that fixation of the ends of the macromolecules on the surface of the filler sharply decreases their mobility, and correspondingly—changes the kinetic parameters of polymerization, particularly, it substantially decreases the rate constant of the bimolecular rupture of the chains. This is the main reason of high steadiness of the process.

In the reaction mixture, a second region appears in the kinetic curves of the polymerization with the increase of the concentration of the monomer, which speaks of the decrease of the rate of the process. Possibly this is conditioned by the accumulation of the polymer on the surface of the ceramic which hinders the accessibility of the molecules of the monomer to the active polymerization centers, e.g., occlusion of the active centers by the macromolecules occurs and the growth rate is controlled by diffusion.

Besides, because of transference of the chain to the monomer—(analogous to blocked polymerization)—the kinetics of the process become salient at some MMA concentration value (20–25% by weight) with possible transition of the radicals from the surface of the filler into the bulk. Presumably, in this case the rate of blocked polymerization is lower than in the initial stages of implanted polymerization. This is because the diffusion retardation—imposed by the filler surface—is less conspicuous. Although, the presented qualitative model of the mechanism and topochemistry of the MMA on the surface of super-conducting ceramic satisfactorily describes the observed peculiarities, nonetheless this topic remains open for further study and discussion.

## 7. Physical-Mechanical Properties of SC Polymer-Ceramic Nanocomposites 

Determination of the physical–mechanical properties of superconducting polymer–ceramic nanocomposites is interesting, not only at ambient but in lower temperatures ranges as well, especially down to the critical transition temperature into the superconducting state. For the superconducting polymer–ceramic nanocomposites, the limiting strength of rupture (*σ*), elasticity modulus (*E*) and elongation (*ε*) were determined at ambient temperatures, whereas for the binder made of SHMPE—at temperatures close 193 K, as well as at the superconducting state temperature of the composite (77 K). The magnitudes of *σ*, *E* and *ε* were determined via distention of the samples at ambient and 77 K temperatures, while at 193 K—under compression conditions. In the latter case the ceramic/binder ratio in the composites was varied as well.

The values of *σ*, *E* and ε are presented in Table 4. Juxtaposition of the durability data for different binders show that the highest rupture resistance and elasticity modulus is observed for polyvinyl alcohol binder and ethylene and tetrafluoroethylene copolymer for practically the same filling index.

**Table 4:** Physical–mechanical properties of the SC polymer–ceramic nanocomposites.

Composition	Weight ratio of the ceramic and the binder	Deformation method	Temperature of deformation (K)	σ, (ΜΠα)	Е, (MPa)	ε,%
**SHMPE+ Y_1_Ba_2_Cu_3_O_6.92_**	85:15	Elongation	300	30	100	10
	85:15		193	60	80	1
	85:15		77	100	-	0.1
**RPE+Y_1_Ba_2_Cu_3_O_6.92_**	80:20	Elongation	300	15	75–80	9.0–10.0
**F-40+Y_1_Ba_2_Cu_3_O_6.92_**	75:25	Elongation	300	32	150	7.2
**i-PB+Y_1_Ba_2_Cu_3_O_6.92_**	80:20	Elongation	300	28	100–110	8.3
**PVA+Irganox+Y_1_Ba_2_Cu_3_O_6.92_**	85:20	Elongation	300	34	130	7.5

**Figure 7:**
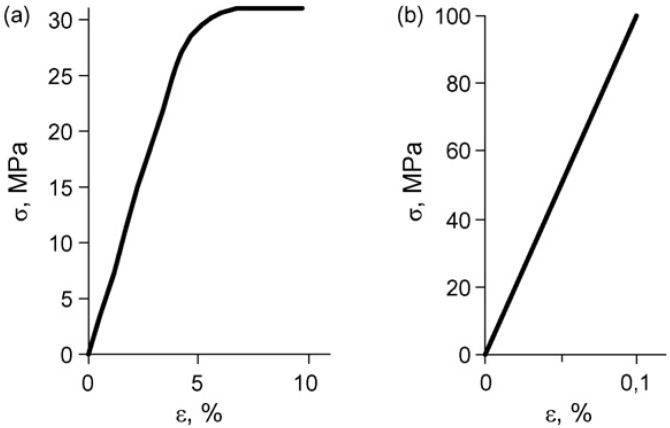
Elongation curves for the Y_1_Ba_2_Cu_3_O_6.97_ nanocomposites with SHMPE, obtained at ambient (a) and in liquid nitrogen (b) temperatures. Ceramic/binder weight ratio: 85:15 [26].

It is noteworthy that at lower usage temperatures of the polymeric materials and filled systems, the durability and elasticity modulus increase while deformity becomes sharply inadequate. Deformation ability is assumed as criterion for workability of the polymeric materials, especially at lower temperatures. That is why the measurements for the composites based on SHMPE were conducted at lower temperatures. Compression durability measurements on the composites based on SHMPE conducted at 193 K show that for the materials containing 90, 85 and 80% weight of ceramic, this index equals 34, 61 and 60 MPa, respectively. Comparison of the rupture properties at ambient and cryogen temperatures seems interesting. Typical diagrams of elasticity for Y_1_Ba_2_Cu_3_O_6.97_ nanocomposites on SHMPE are presented in Figure 7a and b.

It is apparent that for the same filling ratio, at ambient temperatures the samples have a region of high elasticity (Figure 7a), whereas the distention of the samples at 77 K brings about brittle decomposition of the samples in the Hooks deformity region, where the elongation of superconducting polymer–ceramic nanocomposites reaches only 0.1%.

## 8. The Influence of Granulometric Content and Degree of SC-Ceramic Filling on the Physical-Mechanical and SC Properties of Polymer-Ceramic Nanocomposites

In order to understand the process of formation and properties of filled polymeric materials, it is important to investigate their resistance characteristics depending on the average size of particles, the nature of the filler and binder. It is noteworthy that the issue of the stability of the properties of a polymeric binder is disputable if it has a three-dimensional structure. Nevertheless, in [20,24,26] the influence of particle size of the filler and also the nature of the binder on the formation of physical–mechanical properties of the obtained compositions on the basis of epoidal binders is shown.

We will observe the results of our investigations on the influence of the size and concentration of the particles of the filler on the physical–mechanical properties of polymer–ceramic nanocomposites, obtained on the basis of Y_1_Ba_2_Cu_3_O_6.97_ and DR. 

Table 5 shows the influence of the size and filling degree of particles on *ε*, *σ* and *E* and also on the beginning (*T_c_*) and end (*T_f_*) of transition into superconductive state for nanocomposites with cross-linked divinyl rubber.

As it follows from the data of Table 5, the higher the content of the ceramic the higher the rupture strength, the modulus of elasticity and the lower the limiting deformation is, which are independent of the particle size of the Y_1_Ba_2_Cu_3_O_6.97_ ceramic.

The considerable increase in σ and *E* with an increase of the rate of filling evinces the presence of full contact in the polymer-filler interface, and the absence of scaling of the binder from the filler during the deformation of the tested samples. On the absence of scaling also indicate the curves stress *vs.* deformation (Figure 7), where no yield point (point of fluidity) is observed. Indicated facts confirm the earlier conclusion [25,26] about the existence of sufficiently strong interactions between the binder and the surface of the Y_1_Ba_2_Cu_3_O_6.97_ ceramic grains.

This is confirmed by the data on the influence of the ceramics’ grain on *σ* and *E* for the same filling rate as well. Actually, as follows from Table 5, an increase of the mean sizes of the filler decreases the values of *σ* and *E*. This fact can be explained by the diminution of the general contacting surface of the binder with the filler causing the general energy of their interaction to reduce, decreasing consequently the limiting strength and module of elasticity.

It is interesting that with the increase of average size of the ceramic particles, a tendency of increased deformation capacity of composite is observed. Here, most probably, the increase of particle size has a positive effect on the effectiveness of stopping the process of propagation of cracks at high degrees of deforming capacity. In Table 5, there are also data on the influence of average sizes of grains of the ceramic on the critical temperature of the beginning SC transition (*T*_c_) and on its end (*T_f_*).

**Table 5:** The influence of the particle size and filling rate on σ, *E*, ε, *T*_c_, and *T*_f_.

Mean particle size (μm)	Specific surface area (cm^2^/gram)	Filing rate (Mass %)	σ (MPa)	Е (MPa)	ε%	T_c_ (°C)	T_f_ (°C)
		10	1.85	65	280		
**5**	**1132**	20	2.6	85	260		
		30	5.5	115	210		
		40	10	151	160		
		50	17.5	190	100	87	73
		10	1.1	55	277		
**15**	**755**	20	2	74	263		
		30	4.5	100	230		
		40	9	132	183		
		50	17	165	130	91	83
		10	0.8	50	275		
**25**	**453**	20	1.8	68	264		
		30	4	90	225		
		40	7.2	120	200		
		50	15.5	150	157	95	88
		10	0.8	48	272		
**35**	**323**	20	1.6	60	270		
		30	3.5	80	252		
		40	6.2	100	120		
		50	15.5	130	180	95	89

The initial non-fractioned Y_1_Ba_2_Cu_3_O_6.97_ ceramic possesses the following superconducting characteristics: *T*_c_ = 93 K, *T*_f_ = 87 K. Juxtaposition of the initial ceramic of the composite with DR plus different particle size of the ceramic results in dissimilar superconducting properties. For the nanocomposites with mean particle size 5–10 μm, the value of *T*_c_ is lower by 5–10 degrees than for the initial ceramic.

Besides, the transition width (*T*_c_−*T*_f_) widens as well. When the mean particle size becomes more that 20–25 μm, the critical parameters are sustained and even improved *vs.* the initial ceramic. Decreasing the critical temperature and increasing the transition width for the nanocomposites having mean particle size 5–10 μm, are related with two factors:

- Lesser content of the orthorhombic phase in the initial smaller particle size fractions of the ceramic;
- Enhanced utilization of the oxygen in the reactions of thermo oxidation destruction of the binder *vs.* the bigger fractions.

Elevation of the critical temperature of transition into the superconducting state for the nanocomposites obtained from the ceramic with mean particle size 20–25 μm and higher, can be explained, as it has been mentioned earlier [19,23,25] by the intercalation of the elements of macromolecules of the binder into the interstitial layer of the ceramic’s grain.

## 9. Thermo-Physical Properties and Morphological Peculiarities of SC Polymer-Ceramic Nanocomposites

**Figure 8:**
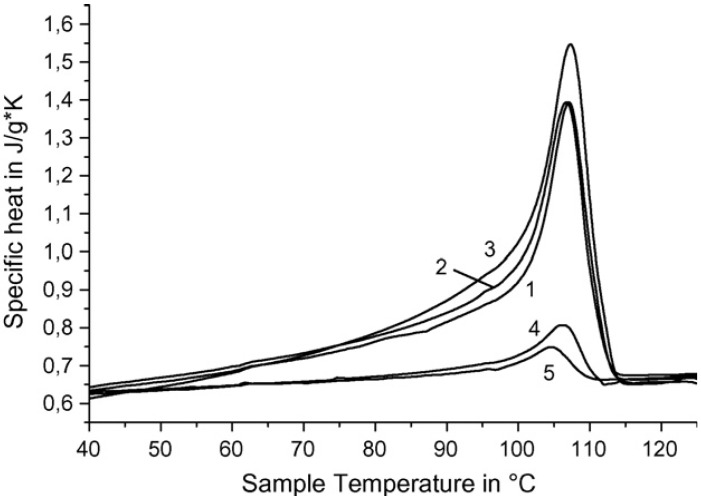
Heat capacity *vs.* temperature for SC polymer–ceramic samples obtained at different initial temperatures (*T*_o_) and with different ratios of ceramics: RPE. *T*_o_ °C —130 (1,4,5), 140 (2), 160 (3); Y_1_Ba_2_Cu_3_O_6.97_:RPE = 90:10 (1–3), 97:3 (4), 99:1 (5) [25].

The dependence of thermal capacity for polymer–ceramic nanocomposites which were obtained on the RPE, is shown in Figure 8. From the curves 1–3 it is seen that the initial temperature of polymer–ceramic superconducting samples’ formation has practically no influence on heat of fusion.

As expected, heat of fusion is changed dramatically depending on the samples’ content. Melting points and enthalpies for SC polymer–ceramic nanocomposites with RPE binder are presented in Table 6. The values were calculated from the experimentally obtained data.

**Figure 9:**
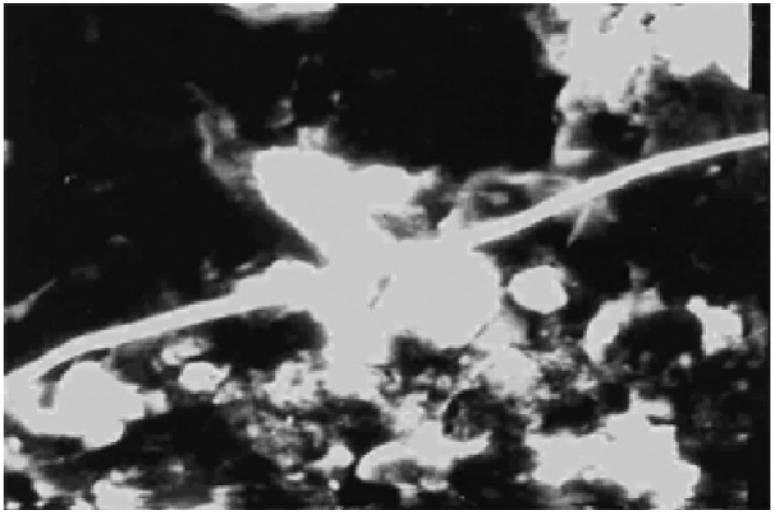
Microscopic photo of Y_1_Ba_2_Cu_3_O_6.97_:RPE = 90:10 SC polymer–ceramic nanocomposites,1 sm= 0.5 μm [25].

The strong increase of Δ*H*_melt_ value (calculated for RPE) could be caused by two reasons. One is that the increase of quantity of SC ceramics leads to an increase of crystallinity. The second and more possible cause is that RPE macromolecules’ fragments intercalation [25,26] into the interstitial layer of ceramics’ grains leads to changes to the crystalline RPE morphology. SC polymer–ceramic nanocomposite samples were investigated by electron microscope. One of the photos of these samples is presented in Figure 9. It is seen that in this case, as for RPE binder, there are fibrils which are not typical for RPE.

**Table 6:** Melting temperatures and enthalpies (∆*H*_m_) of nanocomposites depending on content.

Y_1_Ba_2_Cu_3_O_6,97_: i-PP (mass%)	T_m_ (°C)	ΔH_m_ calc. for 1 g sample (J*g^−1^)	ΔH_m_ calc. for 1 g RPE (J*g^−1^)	Crystallinity (%)
90:10	107	8.4	84	28.6
97:03	107	2.9	97	33
99:01	105	1.3	133	45

Possibly they are the result of intercalation of RPE macromolecule fragments into the layered structure of ceramics. Such a binding presumably influences the mobility of some RPE macro chains and it is logical to assume that crystallization of such macromolecules occurs by cooperative interaction between them.

**Figure 10:**
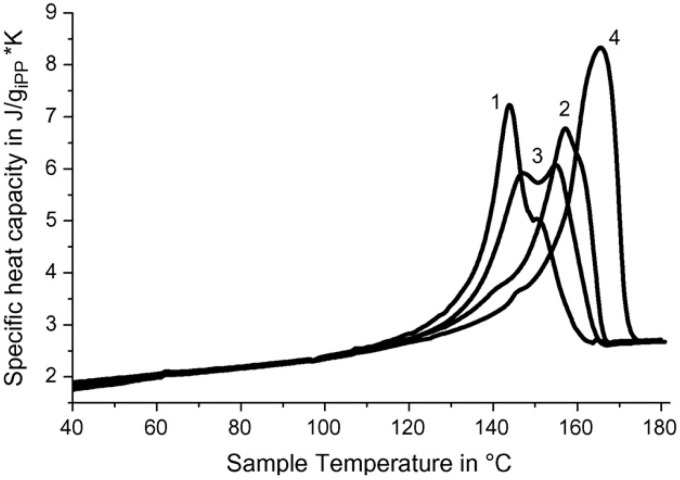
Heat capacity *vs.* temperature for SC polymer–ceramic samples obtained with different ratios of ceramics:i-PP. Y_1_Ba_2_Cu_3_O_6.97_:i-PP = 85:15 (1),70:30 (2), 50:50 (3); pure i-PP (4) [25].

In case of SC polymer—ceramic nanocomposites with i-PP binder, the phenomena observed above are more clear. As it is seen from the dependences of heat capacity from temperature (Figure 10; curves 1, 2, 3), in this case heats of fusion split into two components.

One can assume that this split can be connected with the presence of two different types of structure in the polymer–ceramic nanocomposites. In this case the content of fibrils is appreciably higher in comparison to RPE binder.

## 10. Interphase Phenomena in SC Polymer-Ceramic Nanocomposites

### 10.1. Dynamic Mechanical Properties

**Figure 11:**
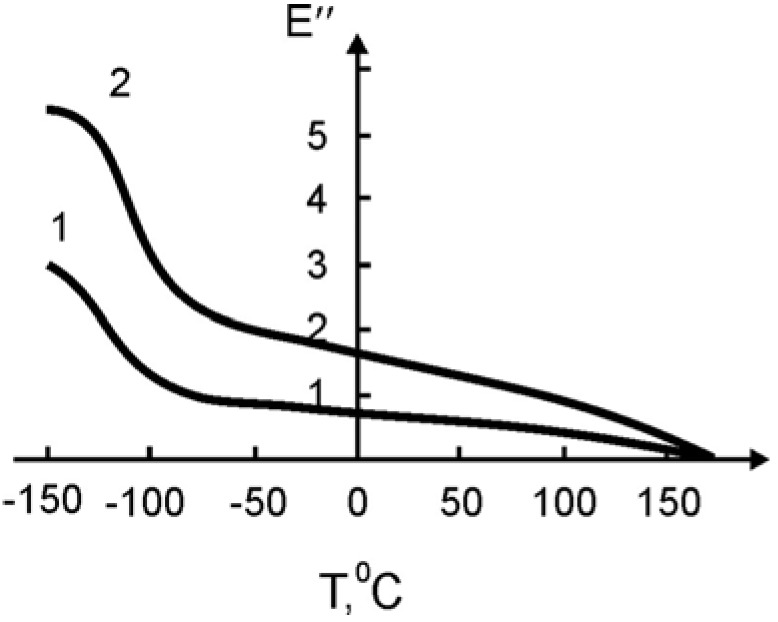
Temperature dependence of *tgδ* for the pure SHMPE and for the SHMPE ceramic nanocomposites. Ceramics content (weight%): curve 1- 0%; 2–15% [25].

**Figure 12:**
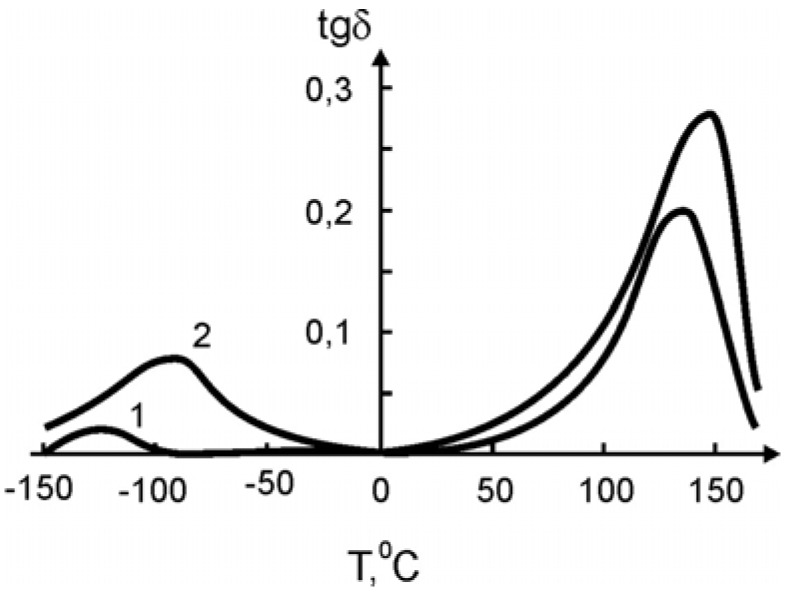
Temperature dependence of *tgδ* for the pure SHMPE and for the SHMPE ceramic nanocomposite. Ceramics content (weight%): curve 1–0%; 2–15% [25].

In Figure 11 and Figure 12 the temperature dependence of the elastic modulus (*E*) and of the loss tangent (*tgδ*), respectively, are shown for the pure SHMPE and the polymer ceramic nanocomposite with 15% filler. Both quantities, *E* and *tgδ*, increase with the increasing amount of ceramic filler.

In both curves two transitions are seen. The step in *E* and the peak in *tgδ* around -100 °C are related to a relaxation process. The broad melting region of the SHMPE from about 50 °C up to 170 °C yields a further softening of the samples and a large peak in *tgδ*. With increasing amount of ceramic filler the peaks in *tgδ* are increased and shifted to higher temperatures, see Figure 12.

It is worth stating that the observed change of the loss tangent curves is a rare feature for conventional non-nanosized ceramic polymer nanocomposites [34]. In [35], increasing mechanical loss peaks are linked with a plate-like structure of the filler. It seems that in this case we have the reverse picture. Some parts of the macromolecules penetrate by intercalation mechanisms into the sandwich structure of the filler, thus creating effects similar to that shown in Figure 12, curve 2.

The increase of the mechanical loss cannot be explained by the agglomeration of the small particles of the filler, because formation of such kind of aggregates can only take place at higher degrees of filling. It seems that the observed increase of mechanical losses is a result of an adsorption of the binder on the surface of the filler and intercalation of fragments of the macromolecules into the interlayer space of the ceramic grains. Such an interaction can change the structure of the polymeric matrix near the boundary of the particles, and—as a consequence—yield an increase of the mechanical losses.

It is known that for some cases the filler shifts the maximum of mechanical losses and *T_g_* towards higher temperatures. It is assumed that the magnitude of the shift is proportional to the surface area of the filler, which explains the polymer-filler interaction.

The non-additive contribution of the added ceramics on the *T_g_* shift (Table 7) points not only to the adsorption interactions, but to the intercalation of the fragments of the macromolecule of SHMPE into the interlayer space of the ceramic grains, as it was indicated above.

**Table 7:** Dynamic mechanical characteristics of the nanocomposites of Y_1_Ba_2_Cu_3_O_6,97_ with SHMPE.

Weight ratio SHMPE: ceramic filler	E′ T =-150°C	E′ T = -100°C	E′ T=25°C	T_c_ °C	tgδ (first trans.)	Tα °C	tgδ (second trans.)
100:0	2.95	1.5	1.1	-127	0.01	134	0.2
85:15	5.1	3.4	1.6	-95	0.06	143	0.25
50:50	10.1	6.5	3.1	-94	0.065	155	—
15:85	—	—	4.5	—	—	157	0.27

It is obvious that such an interaction limits the mobility of the macromolecules, thus changing the packing density of the polymeric chains, and hence a morphology change around the phase boundary may occur. To prove these considerations, the temperatures and enthalpies of melting for the variety of nanocomposites of SHMPE and Y_1_Ba_2_Cu_3_O_6,97_ ceramics were measured directly by differential scanning calorimetry. The obtained data are presented in Table 8.

**Table 8:** Influence of the filling rate on the temperature and enthalpy of melting of the binder in the SHMPE + Y_1_Ba_2_Cu_3_O_6,97_ nanocomposites.

Weight ratio SHMPE: Y_1_Ba_2_Cu_3_O_6,97_	T_melt_ in °C (extrapolated onset of DSC peak)	ΔH_melt_ per gram of SHMPE in J/g	Crystallinity in%
100:0	134	115.0	39.1
85:15	143	116.5	39.7
50:50	155	122.5	41.7
15:85	157	123.5	42.0

With the increase of the filler content in the nanocomposites, the enthalpy of melting recalculated for the polymer fraction (excluding the filler) increases, as shown in Table 8. Again, there is some discrepancy with the curves shown in Figure 11 and Figure 12. In the figures the melting peak shifts to higher temperatures with increasing filler content. This increase of enthalpy is linked either with the overall degree of crystallization or with a change in morphology of the binder at the interphase.

However, based on the obtained results, the dominant role of any of the mentioned factors cannot be definitely proved. For this purpose, it is necessary to conduct thorough electron-microscopic investigations of the nanocomposites samples.

### 10.2. Structural Peculiarities of SC Polymer-Ceramic Nanocomposites

**Figure 13:**
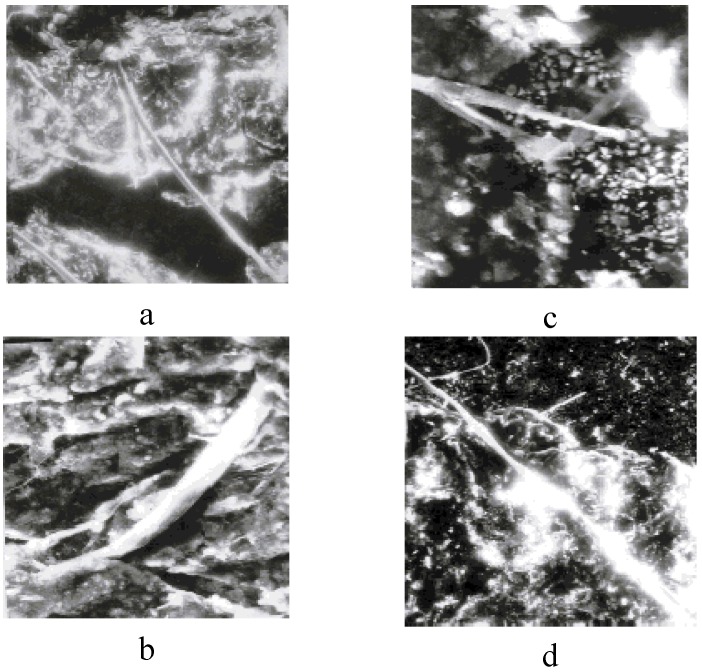
The morphology of polymer –ceramic nanocomposites with SHMPE (a, b) and iPP (c, d) as binders. The nanocomposites - Y_1_Ba_2_Cu_3_O_6,97:_binder = 85:15 (a, c), 80:20 (b, d) weight% . (a, b, c—× 6,000) (d—× 9,000) [25].

**Figure 14:**
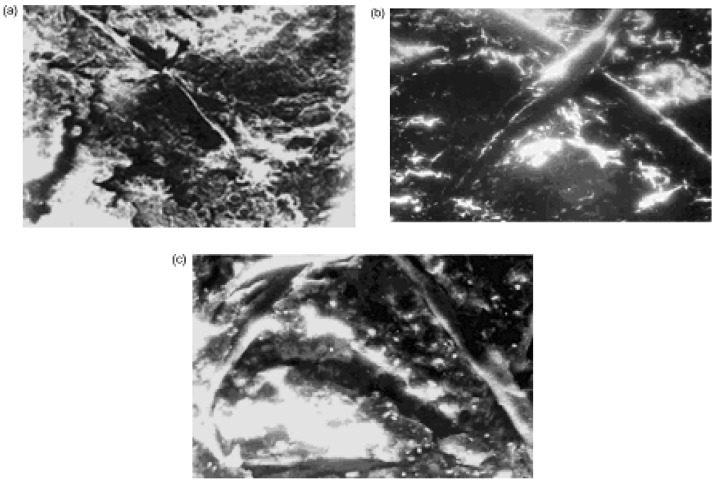
Microscopic photos of polymer–ceramic nanocomposites with different ceramics:i-PP ratios: Y_1_Ba_2_Cu_3_O_6.97_:i-PP =85:15 (a), 70:30 (b), 50:50 (c), 1 sm= 0,8 μm (×8,000) [25].

The investigation of the structures of SC polymer-ceramic nanocomposites by scanning electron microscopy has shown that for both the amorphous and the crystalline polymer matrices, a complete and uniform covering of the ceramic grains by the binder take place (Figure 13a,b,c,d and Figure 14a,b,c).

This indicates a strong interaction at the polymer-ceramic interface. The electron microscopic investigations of polymer–ceramic nanocomposite samples obtained under conditions of variable filling (Figure 14a,b,c) provide support for such an assertion. Besides, fibril structures are observed in the nanocomposites based on crystalline polymers (SHMPE, PP) independent of SC ceramic filler content (Figure 13a,b,c,d and Figure 14a,b,c).

As it is seen from the Figure 14a, in Y_1_Ba_2_Cu_3_O_6.97_:i- PP = 85:15 ratio singular fibrils appear in field of vision of the microscope. With an increase of ceramic additive (Figure 14b and c) the number of fibrils becomes significantly higher.

**Figure 15:**
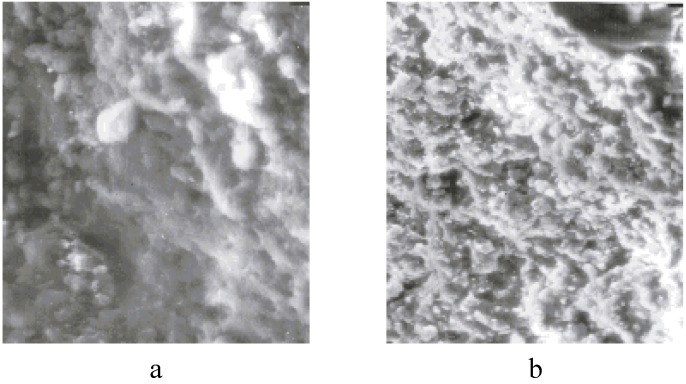
The morphology of the pure polymers SHMPE (a) and iPP (b) (× 8,000) [23].

It should be noted that there are no such fibrilar structures in the initial, unfilled semi-crystalline polymers (Figure 15a,b). These data show that SC ceramic plays a special role during the formation of the crystalline structure of the polymeric matrix during the formation of polymer-ceramic nanocomposites.

## 11. The Influence of the Degree of Filling on Valence State of Copper in Polymer Ceramic Nanocomposites

It is known that high-temperature superconducting oxide ceramics possess their own localized magnetic moments producing a Cu^2+^ EPR signal. Nevertheless, it has to be noted that there are two types of copper atoms in Y_1_Ba_2_Cu_3_O_7-x_ ceramic: Cu^2+^(I) and Cu^2+^(II). The first one is in the CuO chain along the axis direction, while the second is in the CuO_2_ planes along the *ab* plane. For a long time the nature of the Y_1_Ba_2_Cu_3_O_7-x_ ceramic’s EPR response was unclear [36].

Investigation [36,37] of the dependence of Cu^2+^ EPR signal intensity with simultaneous registration of X-ray Absorption Near Edges Structure (XANES) at the Cu^2+^k edges of the same signals [37], as well as Cu^2+^ EPR signal intensity dependence on the substitution degree of Cu^2+^(I) in Y_1_Ba_2_Cu_3_O_7-x_ by Fe atoms showed [37] that EPR signals correspond to the Cu^2+^(I) in the chains and not to the Cu^2+^(II) in the CuO_2_ planes.

**Figure 16:**
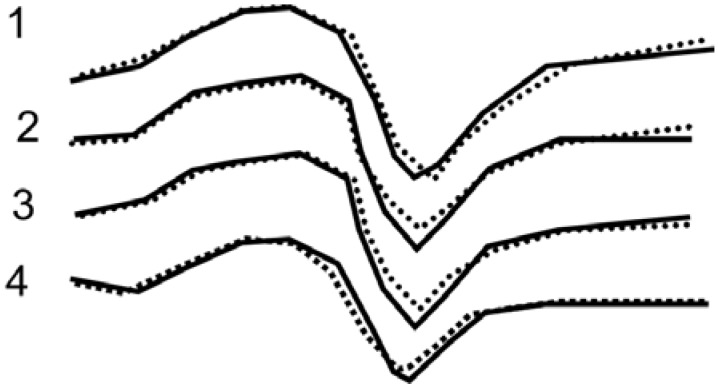
Cu^2+^(I) EPR spectra of SC Y_1_Ba_2_Cu_3_O_7-x_ ceramics (*T_i_*=92.0 K). Reference—dashed line, and nanocomposites (solid line). 1–15% PS and 85% ceramics, 2–15% PMMA and 85% ceramics, 3–20% PE and 80% ceramics, 4–15% copolymer of ST and MMA and 85% ceramics,[24].

Analysis of EPR signals for polymer-ceramic nanocomposites showed that Cu^2+^(I) EPR signals depend on the binder. In Figure16 Cu^2+^(I) EPR spectra are presented both for the Y_1_Ba_2_Cu_3_O_6.97_ and polymer-ceramic nanocomposites with various polymeric binders: polystyrene, polymethylmetacrylate and polyethylene, respectively. These facts demonstrate that the particles of the layered ceramic have nanocomposite structures where the ceramic grains are the precursors of the macromolecules.

Addition of polymer changes the valence state of Cu^2+^(I), as it follows from curves 1-4 of Figure 16. This indicates intermolecular interaction between the Y_1_Ba_2_Cu_3_O_0.67_ ceramic grains and elements of the polymer chains. Such an interaction can be explained by the intercalation of some elements or fragments of the macromolecules into the layered structure of the ceramic. During such an intercalation, superposition of the unpaired electron of the Cu^2+^(I) *3d_x2-y2_* orbital with the orbital of corresponding elements of the polymer chains occurs. As a result, the Cu^2+^(I) EPR response is altered because of the change of the valence state of Cu^2+^(I).

**Figure 17:**
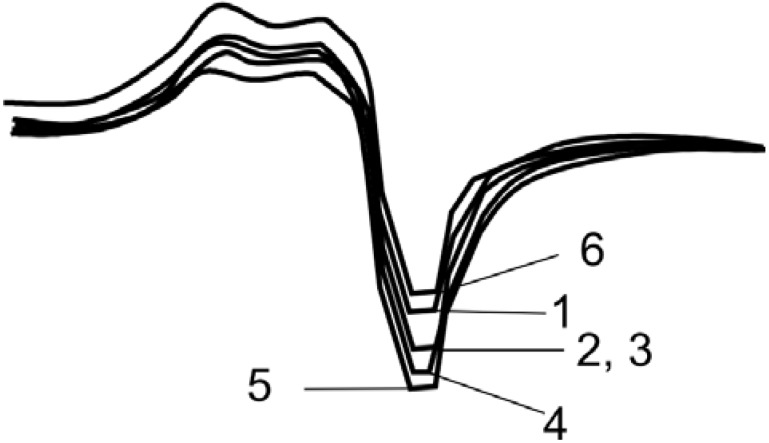
Cu^2+^(I) of the super conducting Y_1_Ba_2_Cu_3_O_6.97_ ceramics (curve 1); and nanocomposites with SHMPE. Curve 2—1% SHMPE; Curve 3—3% SHMPE; Curve 4—5% SHMPE; Curve 5—10% SHMPE; Curve 6—20% SHMPE [24].

Obviously, it is interesting to elucidate whether the intensity of the Cu^2+^(I) EPR signal is dependent on the filling rate, e.g., on the fraction of binder in the ceramic. To answer this question polymer-ceramic nanocomposites were investigated with various Y_1_Ba_2_Cu_3_O_6.97:_SHMPE ratios: 100:0; 99:1; 97:3; 95:5; 93:7; 90:10; and 80:20 (in accordance to their mass %). The obtained data are presented in Figure 17. As it follows from this Figure, curves 1-6, the intensity of the EPR output signal depends on binder content. It is interesting to note that the bigger shift is observed for the smaller quantities of additives (superhighmolecular polyethylene) (curves 2, 3) *vs.* the pure ceramic (curve 1). Further increase of the binder content reduces the signal intensity (curves 4, 5, 6).

## 12. Selection of Mn-, Co-, Zn- and Ni-Containing Metal-Monomer and the Formation of SC Polymer-Ceramic Nanocomposites by Frontal Polymerization in the Presence of Y_1_Ba_2_Cu_3_O_7-x_

Doping of some atoms into a ceramic’s lattice structure is one of the techniques presently used in the search for new SC ceramic nanocomposites in order to enhance the onset of the SC transition temperature. Therefore, using metal-complex polymers as binders is a possible method of regulation of both the critical transition temperature into the superconducting state and its width.

It is known that acrylamide (AAm) complexes of metal nitrates of the first transition group are able to polymerize at frontal regimes. The essence of frontal polymerization is in localized heating of the sample edge initiating the polymerization [28]. The heat evolved is transmitted to neighboring layers by a heat-conductance mechanism, where, in turn, polymerization begins. Thus, the heat wave front propagates over the entire volume. As metal-containing monomers able to polymerize frontally complexes like (AAm)_4_(H_2_O)_2_(MO_3_)_2_, with M = Mn, Co, Y, Cu, *etc.* could be used. A previous investigation showed that frontal polymerization of AAm complexes in the presence of super conducting ceramic is possible only within a limited temperature range.

Experimentally it was shown that the lower temperature limit for carrying out the reaction (100 °C) is given by the stability of frontal polymerization upon propagation of vertical heat waves from up to down. This sharply reduces and limits the yield of nanocomposites. The upper temperature limit (200 °C) is determined by the thermal degradation of nitrate groups in the nanocomposites obtained.

**Figure 18:**
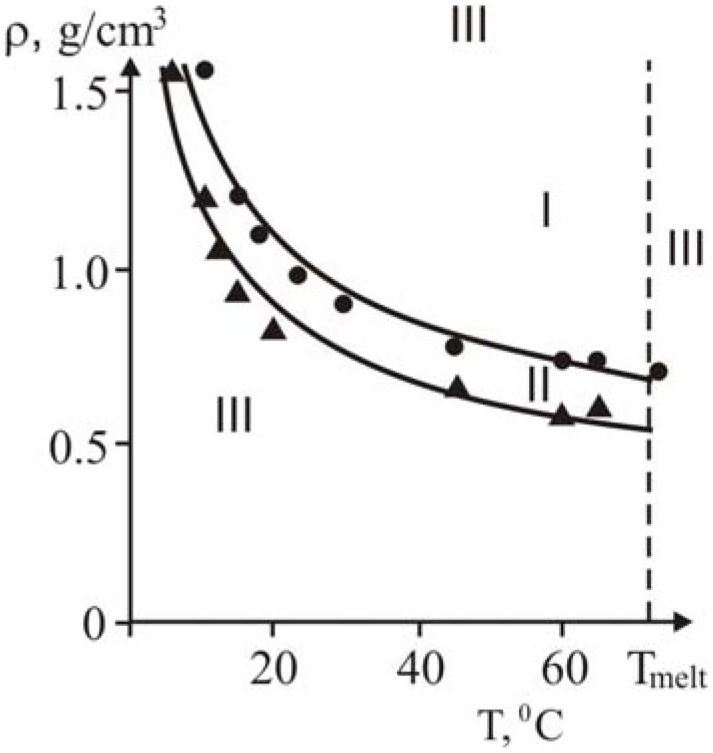
Density-temperature diagram for three regimes of frontal polymerization [28].

The influence of density, temperature and reactor diameter on the structure of heat waves, rate of front propagation, range of existence of steady-state regimes was investigated. Experimental results obtained at different temperatures and densities are summarized in Figure 18.

Here, curve 2 is the domain of steady-state heat polymerization waves which is limited by the straight line corresponding to limiting packing of reaction media (*ρ_lim_*) and full melting of crystalline monomer. There are no wave regimes of frontal polymerization below curve 1, Figure 18, as well as when *ρ >ρ_lim_* and *T ≥ T_melt_*.

For mass ratios of the ceramic:metal polymer ≤ 80:20, addition of the minute quantities of Y_1_Ba_2_Cu_3_O_7-x_ ceramic does not allow frontal waves to travel from up to down up to 100 °C. At the same time, the formation of the propagating frontal regimes of heat waves is observed at temperatures above 60 °C, when the wave is initiated from the bottom and the front propagates from bottom to up.

**Table 9:** SC characteristics of the polymer-ceramic nanocomposites with Mn, Co, Ni, Zn metals.

Formula of the composition				Nature of the metal	T_c_ (K)	T_f_ (K)
Y_1_Ba_2_Cu_3_O_6,98_		Metal-monomer complex				
gr.	mass(%)	gr.	mass(%)			
0.293	43	0.388	57	Mn	95	87
0.396	50	0.396	50	Mn	94	85
0.90	70	0.29	30	Mn	94	84
0.518	73	0.196	27	Mn	93	85
0.90	70	0.39	30	Mn	95	85
0.416	67	0.209	33	Mn	94	83
0.552	78	0.156	22	Mn	95	85
0.325	51	0.318	49	Co	93	83
0.432	60	0.283	40	Co	92	84
0.503	70	0.228	30	Co	92	84
0.486	70	0.208	30	Ni	95	83
0.552	78	0.156	22	Zn	95	86
0.416	67	0.209	33	Zn	94	83

This situation is explained by the gas evolution during frontal polymerization, as well as by the inhibition of some constituents of the evolved gases on the process of polymerization.

The results of the investigation of super conducting properties of Mn-, Co-, Ni-, Zn-containing polymer-ceramic nanocomposites are presented in Table 9. As it follows from the data shown in Table 9, the onset of the transition into the SC state (*T_c_*) is shifting towards higher temperatures compared to the initial ceramic, *T_c_* = 93 K, *T_f_* = 78 K. The SC onset increase reaches 1–3 K for *T_c_* and more than 5 K for *T_f_*. It is known from the literature that the Y_1_Ba_2_Cu_3_O_7-x_ high-temperature SC ceramic exhibits an anti-ferromagnetic transition, e.g., transition into the spin glassy (vitrous) state before the transition into the SC state. Presumably, anti-ferromagnetic and high-temperature SC states are co-existing. Taking into account that Co, Ni, Mn are known as anti-ferromagnetic metals, which intercalate into the interstitial layers of the ceramic, causing the 3-D properties of the ceramic grains to change. That is why *T_c_* increases and the transition width decreases. This interpretation is supported by our data on the change of the valence state of Cu in polymer-ceramic nanocomposites.

## 13. Aging of SC Polymer-Ceramic Nanocomposites

The prevailing orientation of the crystallites on the (110) direction in the Y_1_Ba_2_Cu_3_O_6.92_ sample is practically stable over time and is strong in absolute value intensity line. Y_1_Ba_2_Cu_3_O_6.97_ specimen is oriented along (006) and this reflex is not changed in absolute value as the time passed on. Besides, Y_2_BaCuO_5_ semiconductor phase is identified (211)—2% in content. The texture in both of these specimens is controlled by the relative intensities of the peaks: (110), (103), (005), (014), (113), (006), (020), (200).

As it turns out, the superconducting properties of Y_1_Ba_2_Cu_3_O_6.97_ specimens are stable enough over time (Table 10), while some of the insignificant property changes are reversible, and can be ascribed to auto oscillating processes, which are typical for structurally unstable electronic systems in solids, and can’t be attributed to the aging of the structure. It seems, that the Y_2_BaCuO_5_ semiconductor phase in Y_1_Ba_2_Cu_3_O_6.97_ is the oxygen transport phase [38], being present in definite amounts in high-temperature super conducting ceramic, supporting the stabilization of the super conducting characteristics on behalf of vacant positions of the oxygen. 

The some increasing of the *T_c_* value, immediately after polymer-ceramic nanocomposites formation, is linked with intercalation of the fragments of the macromolecules binders into interlayer space of ceramics grains during hot pressing process [25,26].

The change of the superconducting properties is dependent on the chemical composition of the polymeric binder as the kinetic investigations of the aging process of the SC polymer-ceramic nanocomposites show. The superconducting characteristics of the ST-MMA copolymers are worse, whereas for the polyethylene polymer - improvement is observed.

Increase of the critical temperature of transition into the superconducting state for the nanocomposites with polyethylene binder, is possibly linked with the processes taking place after the stage of formation of polymer-ceramic nanocomposites.

In our previous works the presence of interaction forces between the elements of the polymeric binder with the surface of the ceramic grains up to intercalation of these elements into the interlayer space of the ceramics’ particles was shown.

As it is evident from the data of the Table 10, one can assume that interaction of the elements of the polymeric binder with the surface of ceramic’s grain through the intercalation mechanism, although slow, nevertheless proceeds at ambient temperatures. For this reason some increase of *T_c_* value is observed (1–1.5 degrees).

As already stated, for the superconducting nanocomposites of ST with MMA copolymers, the aging process brings a decrease of *T_c_* by 1–1.5 °C, an increase of Δ*T_c_* and decrease of *η*. Presence of antioxidant (NG-2246) does not influence the stated characteristics. This kind of stated characteristics changes can be explained by the enhanced inclination of the ST-MMA copolymer binders towards destruction under the impact of ultraviolet radiation.

**Table 10:** Super conducting characteristics of the high-temperature super conducting ceramic (Y_1_Ba_2_Cu_3_O_6.92_, Y_1_Ba_2_Cu_3_O_6.97_), and of the polymer-ceramic nanocomposites based on Y_1_Ba_2_Cu_3_O_6.97_. The super conducting transition temperature (*T_c_*, K), the transition width (Δ*T_c_*, K), and the orthorhombic distortion of the lattice structure - ((*b* − *a*)/*a*,*η*).

Time, Months	Super conducting characteristics of the specimens	Y_1_Ba_2_Cu_3_O_6,.97_	Type of polymeric binder in the composition (the ratio of the Y_1_Ba_2_Cu_3_O_7-x_:polymer component = 85:15%)			
			PE	PS+NG-2246	ST:MMA (40:60 mol%)	SPL (ST:MMA) (80:20 Mole%) + NG-2246
0	η	0.0189	0.0185	0.0197	0.0194	0.0202
	T_c_	92.0	91.8	93.0	92.6	93.4
	ΔT_c_	6.5	6.5	7.0	6.5	9.0
6	η	0.0185	0.020	0.0185	0.0185	0.0185
	T_c_	91.7	92.8	91.7	92.1	92.2
	ΔT_c_	6.0	6.0	≈ 7	≈ 7	≈ 7
12	η	0.0185	0.0196	0.0181	0.0180	0.0180
	T_c_	91.8	92.8	91.7	92.0	92.0
	ΔT_c_	≈ 8	≈ 8	≈ 9	≈ 8	≈ 9

For obtaining more substantial data on the elevation of *T_c_* during the aging of the polymer-ceramic nanocomposites based on the polyethylene matrix, similar investigations were carried out on the samples obtained by the gas-phase polymerization of ethylene in the presence of Y_1_Ba_2_Cu_3_O_6.97_ ceramics as well. 

## 14. The Preparation of SC Polymer-Ceramic Nanocomposites by Gas-Phase Polymerization of Ethylene

Catalytic properties of oxide ceramic-perovskites have been known for a long time [39,40,41]. Naturally here a question is posecd over the issue of catalytic activation of the ceramic’s surface for polymerization of gas-phase monomers (ethylene, propylene, *etc.*) without using polymerization catalysts on the surface.

Presumably, the layered structure of the crystalline orthorhombic phase will allow coordinating ethylene (or other kinds of olefins) on the surface of the Y_1_Ba_2_Cu_3_O_7-x_ ceramic’s grains and, in this case, the polymerization process may proceed in the presence of polymerization (alkyl-aluminum) co-catalysts.

**Figure 19:**
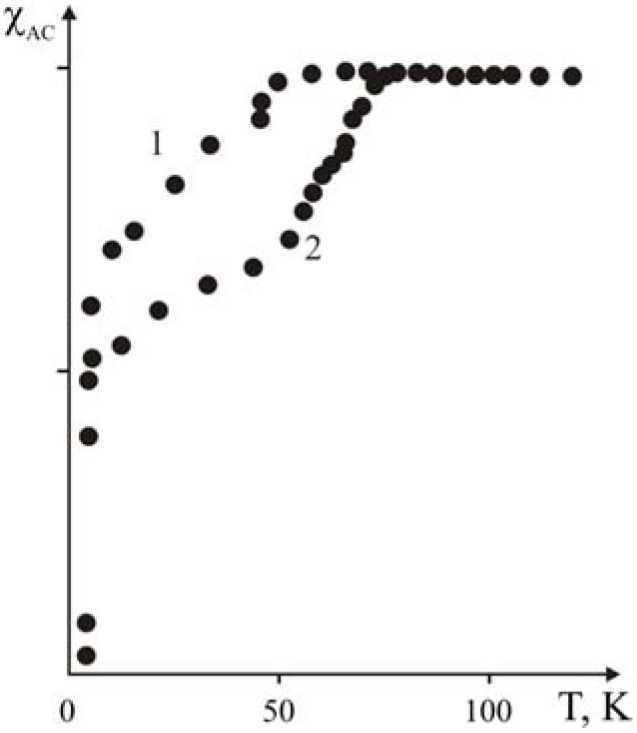
Dependence of the critical temperatures of transition into super conducting state and the width of (T_c_–T_f_) on the weight ratio of the ceramic and polyethylene, [21].

It should be noted that in this case the quantity of forming polymer was regulated by the time of polymerization permitting preparation of nanocomposites with various binder contents. In this case there is the Meisner-effect for all samples without addition of antioxidants. It is explained by decreasing diffusion velocity of oxygen located on the surface of ceramic’s grains because of formation of the polymer covering around the oxide ceramic’s particles. The parallel displacement of curves of the SC transition towards enhancement of the *T_c_* from 46 K (for initial ceramics) up to 70 K (Figure 19) with increasing polyethylene content is observed.

The decrease of *T_c_* for initial Y_1_Ba_2_Cu_3_O_6.97_ ceramics from 92 K up to 46 K and accordingly for the obtained nanocomposites are possibly linked with high temperature activation of the surface of the ceramic’s grains.

Three transition temperatures into the super conducting state were detected on the curves (Figure 19) of superconducting transition for all of the nanocomposites: low temperature (*T_l.c_*_._~5 K), which does not depend on the formula of the composition, mid-temperature (*T_m.c_*), and comparatively high temperature (*T_h.c_*), which depend appreciably on the content of polyethylene in the nanocomposites. That is why three transition temperatures were indicated in the Table 11, *T_l.c_ T_m.c_ T_h.c_* accordingly.

A different degree of degradation of various fractions of the ceramic cause the transition into the super conducting state have a stepwise character and, accordingly, thus decreases the rate of intercalation in relation with the degree of amorphizition of the ceramic’s grain.

It is interesting to note that the beginning of the high temperature transition (*T_h.c._*) as well as its width substantially depends upon the weight ratio of the components of the composition. As was already stated, the observed increase of the (*T_h.c._*) and (*T_h.c_–T_h.f._*), can be explained by the sufficient intercalation (see the former quarterly and annual report) of the separate fragments of the polyethylene or the co-catalyst - into the interstitial layer of the Y_1_Ba_2_Cu_3_O_6.97_ ceramic during the gas phase polymerization of the ethylene.

**Table 11:** Dependence of the critical temperatures of transition into super conducting state and the width of (*T_c_*–*T_f_*) on the weight ratio of the ceramic and polyethylene.

Ceramic:polyethylene weight ratio	T_l.c.,_K	T_l.f.,_K	T_m.c.,_K	T_m.f.,_K	T_h.c.,_K	T_h.f.,_ K
100:0	10	4.5	~30	~10	46	~30
80:20	10	4.5	~50	10	70	~50
50:50					79	~63

**Figure 20:**
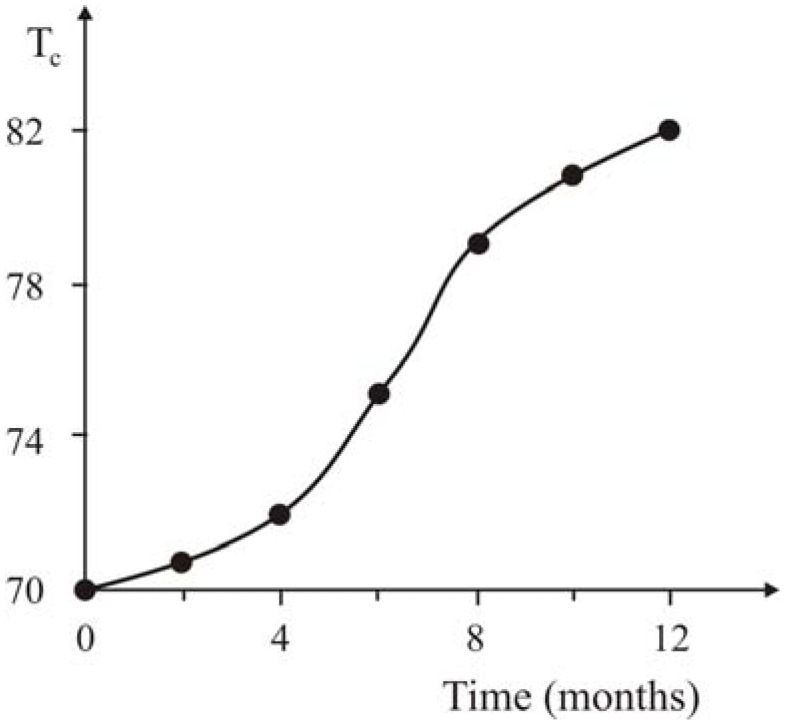
The aging kinetics of SC nanocomposites with 20% content PE, [21].

As in previous experiments the SC nanocomposites containing 20 wt.% PE was kept in air at ambient temperatures in the course of year. The measurement of the SC characteristics in six and twelve months has shown an increase in *T_c_* by 5 K and 12 K correspondingly (Figure 20).

These results confirm our supposition that even at ambient temperatures, the process of intercalation of the fragments of macromolecules into layered structure of grains of the ceramics takes place (albeit slowly), leading to formation of local nano-compositions.

## 15. Aging of the SC Characteristics of Polymer-Ceramic Nanocomposites with Mn, Co, Zn, Ni, Metals 

Presently, doping of some atoms into the ceramic’s lattice structure is one the ways of searching for new SC ceramic nanocomposites with enhanced SC transition temperature onset. Therefore, use of metal-complex polymers as binders is a possible method of regulation of both the critical transition temperature into the super conducting state and its width [21].

It is known that acrylamide (AAM) complexes of first transition row metal nitrates are able to polymerize at frontal regimes. The essence of frontal polymerization is in localized heating of the sample edge initiating the polymerization. The heat evolved (by the heat-conductance mechanism) is transmitted to neighboring layers, where the polymerization begins. The heat wave front propagates throughout the entire volume. Complexes of formula (AAM)_4_(H_2_O)_2_(MeO_3_)_2_ (where Me = Mn, Co, Zn, Cu) are used as metal-containing monomers for frontal polymerization experiments. Investigations showed that frontal polymerization of AAM complexes in the presence of superconducting ceramic only occurs in a definite temperature range. It was shown that the lower temperature limit (100 °C) of the reaction is due to the stability of the polymerization front upon propagation of vertical heat waves from up to down. This sharply reduces and limits the output of nanocomposites. The upper temperature limit (200 °C) is determined by heat degradation of nitrate groups in the nanocomposites obtained.

**Table 12:** The effect of aging on super conducting characteristics of the polymer-ceramic nanocomposites with Mn, Co, Zn, Ni, metals.

Formula of the composition				Metal	Pressing duration, min.	T_c_/T_c_ (K)	T_f_/ T_f_ (K)
Y_1_Ba_2_Cu_3_O_6.98_		Metal-monomer complex					
gr	mass (%)	gr	mass (%)				
0.293	43	0.388	57	Mn	10	95/96	87/89
0.396	50	0.396	50	Mn	5	94/96	85/87
0.90	70	0.29	30	Mn	5	94/95	84/88
0.518	73	0.196	27	Mn	5	93/96	85/90
0.90	70	0.39	30	Mn	5	95/96	85/91
0.416	67	0.209	33	Mn	10	94/96	83/86
0.552	78	0.156	22	Mn	5	95/96	85/88
0.325	51	0.318	49	Co	5	93/95	83/85
0.432	60	0.283	40	Co	5	92/9	84/89
0.503	70	0.228	30	Co	2	92/95	84/87
0.486	70	0.208	30	Ni	5	95/96	83/85

Up to 100 °C, addition of minute quantities of Y_1_Ba_2_Cu_3_O_7-x_ ceramic (for mass ratio of the ceramic: metal polymer ≤ 80:209) does not allow frontal regimes to travel from top to bottom. At the same time, the formation of propagating frontal regimes of heat waves is observed at temperatures above 60 °C, when the wave is initiated from the bottom and the front propagates from bottom to up. This situation is explicable by the gas evolution during frontal polymerization, as well as by the inhibition of some constituents of the evolved gases on the polymerization process.

The results of the investigation of super conducting properties of Mn, Co, Zn, Ni-containing polymer-ceramic nanocomposites are given in Table 12. As it follows from this data, initiation of the transition into superconducting state (*T_c_*) is shifted towards higher temperatures, compared to the initial ceramic of *T_c_* = 93 K, *T_f_* = 78 K. This *T_c_* onset elevation amounts to 1–3 degrees, and *T_f_* —more than 5 degrees.

It is known from literature that Y_1_Ba_2_Cu_3_O_7-x_ high-temperature super conducting ceramic undergoes an anti-ferromagnetic transition into a spin glassy state before the transition into the superconducting state. Moreover, it is presumed that anti-ferromagnetic and high-temperature superconducting states are co-existing. Taken into account that Co, Mn, Zn, Ni, are known as anti-ferromagnetic metals, which intercalate into the interstitial layers of the ceramic, this causes the 3-D properties of the ceramic’s grain to change. That is why *T_c_* elevates and transition width decreases. This explanation is supported by our data on the change of valence state of Cu in polymer-ceramic nanocomposites.

Samples of metal containing SC polymer-ceramic nanocomposites with various metal polymers were investigated on aging. The results of measurements of SC characteristics of nanocomposites before and after aging in the course of 1 year are given in Table 12 It follows that during aging of Mn, Co, Zn, Ni-containing polymer-ceramic nanocomposites, *T_c_* increases by 1–3 K while SC transition width narrows down of to 4–6 K. There are no reasonable explanations for this fact.

## 16. Conclusions

In conclusion, it can be stated that superconducting characteristics of the polymer–ceramic composites are determined by two main competing factors:

- Interaction of the elements of the macromolecule of the binder with the surface of the ceramic with further intercalation of these elements into the interstitial layer of the ceramic, thus causing some elevation of *T*_c_ (by 1–3 K) of the composites versus the initial ceramic.
- Stability of the polymeric binder against thermo-oxidative destruction, and, possibly, by the mechanism of disintegration of macromolecule (e.g., by the composition of the products evolved upon destruction of the matrix). The possible participation of the oxygen from the orthorhombic phase of the ceramic in the processes of thermo-oxidative destruction of the matrix conditions broadening of the temperature interval of superconducting transition.
- Thus, on the basis of the obtained data in on the nature of the change of physical–mechanical and SC properties of the high temperature superconducting nanocomposites depending on granulometric composition and filling degree, it can be concluded that the peculiarities of forming the boundary of interstitial layer, oxide highly conductive ceramic–binder, its structure and adhesion of the binder with the surface of the ceramic play a significant role in the formation of superconducting and physical–mechanical properties of the obtained nanocomposites. For the nanocomposites mentioned an increase of melting heats has been found. This can be explained by intercalation of fragments of the macromolecules into the interstitial layer of the ceramics’ grains. This phenomena leads to the change of the morphological structure of the superconducting nanocomposites obtained. The latter has been proved by electron microscopy.
- The ceramic-binder boundary plays an important role in superconductive and mechanic properties of SC polymer ceramic nanocomposites based on SHMPE. Thus, according to the data on dynamic mechanical properties, obtained over a wide temperature interval, it can be concluded that the peculiarities of the formation of the interface within the ceramic-binder boundary is most important. The data on electron microscopy and EPR signals on Cu^2+^ (1) are sound proof of the presence of intercalated fragments of the macromolecules into the interlayer space of the ceramic grains, leading to the formation of nanostructures.
- Thus, the activation of Y_1_Ba_2_Cu_3_O_6.97_ ceramics grains’ surface allows using them as catalysts of olefin gas phase polymerization. Metal monomer frontal polymerization at the presence of Y_1_Ba_2_Cu_3_O_6.97_ ceramics allows preparation of metal containing polymer-ceramic SC nanocomposites.

The results of SC polymer-ceramic nanocomposites aging show that at room temperatures the intercalation of macromolecules fragments which are binding the interlayer space of ceramics takes place. This process results in formation intercalated nanostructures in the grains of ceramics.
